# Whipple's Disease: Our Own Experience and Review of the Literature

**DOI:** 10.1155/2013/478349

**Published:** 2013-06-17

**Authors:** Jan Bureš, Marcela Kopáčová, Tomáš Douda, Jolana Bártová, Jan Tomš, Stanislav Rejchrt, Ilja Tachecí

**Affiliations:** 2nd Department of Medicine-Gastroenterology, Charles University in Praha, Faculty of Medicine at Hradec Králové, University Teaching Hospital, 50005 Hradec Králové, Czech Republic

## Abstract

Whipple's disease is a chronic infectious systemic disease caused by the bacterium *Tropheryma whipplei*. Nondeforming arthritis is frequently an initial complaint. Gastrointestinal and general symptoms include marked diarrhoea (with serious malabsorption), abdominal pain, prominent weight loss, and low-grade fever. Possible neurologic symptoms (up to 20%) might be associated with worse prognosis. Diagnosis is based on the clinical picture and small intestinal histology revealing foamy macrophages containing periodic-acid-Schiff- (PAS-) positive material. Long-term (up to one year) antibiotic therapy provides a favourable outcome in the vast majority of cases. This paper provides review of the literature and an analysis of our 5 patients recorded within a 20-year period at a tertiary gastroenterology centre. Patients were treated using i.v. penicillin G or amoxicillin-clavulanic acid + i.v. gentamicin for two weeks, followed by p.o. doxycycline (100 mg per day) plus p.o. salazopyrine (3 g per day) for 1 year. Full remission was achieved in all our patients.

## 1. Introduction

Whipple's disease is a rare, chronic, and infectious systemic disease caused by the bacterium *Tropheryma whipplei*, a member of the diverse order of *Actinomycetales*, usually found in soil [1].

## 2. History

In 1907, George Hoyt Whipple described a case of a 36-year-old physician (medical missionary) [2]. Whipple published this report only two years after his graduation from the Johns Hopkins University in 1905. Subsequently he won the Nobel Prize in physiology and medicine (for his “discovery concerning liver therapy of anaemia” in 1934).

Whipple described his case as “gradual loss of weight and strength, stools consisting chiefly of neutral fat and fatty acids, indefinite abdominal signs, and a peculiar multiple arthritis” [2]. The patient died of this progressive illness. Whipple called it intestinal lipodystrophy since he observed the accumulation of  “large masses of neutral fats and fatty acids in the lymph spaces.” The illness was renamed Whipple's disease in 1949 [3]. An infectious aetiology was suspected as early as Whipple's initial report. Whipple described a “number of rod-shaped organisms resembling in form the tubercle bacillus” in the vacuoles of the foamy cells [2]. Until the early 1960s, the disease was considered to be a uniformly fatal and untreatable primary disorder of fat metabolism. In 1952, the first successful treatment with antibiotics was reported (a prolonged period of chloramphenicol) [4]. Bacillary bodies were identified by transmission electron microscopy, establishing the bacterial cause in 1961 [5]. No name was given to the organism until 1991 when the nomenclature was proposed. On the basis of this gene sequence, the organism was classified as an actinobacterium and successfully was cultivated in vitro in HEL cells for the first time in 1999 [6]. The name of this bacterium—*Tropheryma whipplei*—is derived from Greek “trophe” (nourishment and food) and “eryma” (fence and barrier). 

Several original tissues obtained from the autopsy performed by Whipple were still available and were reviewed. Immunodetection of *Tropheryma whipplei* was successful, even nearly 100 years later after the initial description [7, 8]. 

## 3. Epidemiology

Whipple's disease is extremely rare illness. Between 1907 and 1987 there were 696 reported cases only, and the annual incidence since 1980 has been approximately 30 cases per year worldwide. The disorder has a predilection for white males of European ancestry (less than 3% are Africans or other ethnic groups), suggesting an underlying genetic predisposition [3]. The disease most commonly started between 40 and 60, with a mean age at onset of 50 years. The male-to-female ratio is about 8 : 1. Whipple's disease occurs more commonly among farmers (35%) and other subjects in contact with soil or animals. Either direct person-to-person transmission or nosocomial transmission (e.g., by gastrointestinal endoscopy) has not been documented [9]. 

*Tropheryma whipplei* DNA has been reported in 1%–11% of stool specimens from the healthy adult population of people in Europe [10–12] and in wastewater samples, supporting the hypothesis that the bacterium is a soil or water organism [13, 14]. Chronic asymptomatic carriage of *Tropheryma whipplei* was proved by culture from stool and saliva (ranging from a prevalence of 4% in the control group to 12% among a subgroup of sewage workers) [15]. Fenollar et al. [16] conducted serologic and molecular studies, including genotyping, on saliva, faeces, and serum from 74 relatives of 13 patients with classic Whipple's disease. They detected *Tropheryma whipplei* in 24 (38%) of 64 faecal samples and 7 (10%) of 70 saliva samples from relatives but found no difference between persons related by genetics and marriage. Seroprevalence was higher among relatives (23/30; 77%) than in the general population (143/300; 48%). The high prevalence of *Tropheryma whipplei* within families suggests intrafamilial circulation [16].

## 4. Pathogenesis

The pathogenesis of Whipple's disease still remains obscure [3, 17, 18]. Invasion or uptake of the bacteria is widespread throughout the body, including the intestinal epithelium, macrophages, capillary and lymphatic endothelium, liver, brain, heart, lung, synovium, kidneys, bone marrow, and skin. All of these sites show a remarkable lack of inflammatory response to *Tropheryma whipplei*. In addition, the organism exerts no visible cytotoxic effects upon host cells, thereby allowing massive accumulation of *Tropheryma whipplei* at sites of infection [3]. Host factors rather than the genotype of the bacterium influence the course of infection. The incidence of Whipple's disease is very low despite the ubiquitous presence of *Tropheryma whipplei* in the environment. Therefore, it has been suggested that host factors indicated by immune deficiencies are responsible for the development of Whipple's disease. In patients with Whipple's disease, peripheral T cell proliferation is reduced after stimulation with phytohemagglutinin and concanavalin A but patients have normal levels of immunoglobulins, suggesting a specific defect in cell-mediated immunity [19]. Moos et al. [19] showed reduced or absent *Tropheryma whipplei*-specific Th1 responses, whereas their capacity to react to other common antigens like tetanus toxoid, tuberculin, *Actinomycetes*, *Giardia lamblia*, or cytomegalovirus was not reduced compared with controls. Hence, an insufficient *Tropheryma whipplei*-specific Th1 response may be responsible for an impaired immunological clearance of *Tropheryma whipplei* in patients with Whipple's disease [19]. Functional Th2 responses, characterised by enhanced expression of interleukin 4, are increased [20]. Deactivation of macrophages by interleukin 4 is required for growth of *Tropheryma whipplei* in cell culture [21]. Antigen presentation by the MHC class II is absent or diminished on the intestinal epithelial cells of patients with active Whipple's disease. These findings normalise with treatment [22]. Taken together, these observations suggest underlying host immune deficiency and possibly secondary immune downregulation induced by the bacterium [3]. 

## 5. Clinical Features 

### 5.1. Symptoms

Nondeforming arthritis is most characteristic of Whipple's disease and is frequently the initial symptom. Joint complaints might precede by many years the onset of intestinal symptoms (by a mean of six years). When gastrointestinal symptoms occur, the disease usually progresses rapidly with marked diarrhoea, abdominal pain, weight loss, muscle wasting, weakness, anorexia, dyspnoea, cough, headache, low-grade fever, nonthrombocytopenic purpura, and symptoms of anaemia and deficiencies of vitamins. Besides arthritis, other extraintestinal symptoms might occur, like pulmonary hypertension, congestive heart failure, “culture-negative” endocarditis, polyserositis (pericarditis, pleural effusion, and ascites), metabolic bone disease, cognitive disorders (including dementia) and neurological signs (cerebellar ataxia, oculomasticatory myorhythmia, oculo-facial-skeletal myorhythmia, and others) [3, 23–35]. 

In a large Spanish analysis of 91 cases of Whipple's disease from 1947 to 2001 [35], the most common symptoms and signs were weight loss (80%), diarrhoea (63%), lymphadenopathy (35%), skin signs (32%), abdominal pain (27%), fever (23%), joint complaints (20%), and neurological symptoms (16%). Arthralgias, diarrhea, and fever were noted prior to diagnosis in 58, 18, and 13% of patients, respectively. Intestinal biopsy was positive in 94%. There were nine relapses, four of which were neurological, although all occurred before the introduction of cotrimoxazole [35]. In a French series of 52 patients from 1967 to 1994 [24], clinical manifestations preceding the diagnosis were articular for 35 patients (67%), digestive for 8 patients (15%), general for 7 patients (14%), and neurologic for 2 patients (4%). At a later stage of the disease, 44 patients (85%) were presented with diarrhoea, weight loss, and malabsorption, while 8 patients (15%) did not show any gastrointestinal symptom throughout the development of the disease. Forty-three patients (83%) were presented with arthralgia or arthritis, and 11 (21%) had prominent neurologic symptoms. In addition to this, cardiovascular symptoms were present in 9 patients (17%); mucocutaneous symptoms in 9 patients (17%); pleuropulmonary symptoms in 7 patients (13%); and ophthalmologic symptoms in 5 patients (10%). With treatment, the disease evolved favourably in 47 patients (90%), while 5 patients (10%) had unfavourable outcomes (2 deaths from neurologic involvement, 1 patient with chronic dementia, and 2 patients with digestive symptoms insensitive to antimicrobial therapy). Of the 41 patients initially treated successfully and whose treatment has been completed, clinical evolution after discontinuation of treatment was favourable in 34 cases (83%). Clinical relapses occurred in 7 patients. No relapse was observed after treatment using trimethoprim sulfamethoxazole, alone or following a combination of penicillin and streptomycin, or after the combination of penicillin and streptomycin, whatever the oral follow-up treatment prescribed [24]. 

Less-common symptoms include fever and skin hyperpigmentation. Hyperpigmentation may occur as a consequence of vitamin D malabsorption, which may induce compensatory secondary hyperparathyroidism leading to enhanced MSH and ACTH production [36]. In addition to this, *Tropheryma whipplei* infection may induce hypothalamic dysfunction and adrenal gland insufficiency [37]. Malabsorption of vitamin B12 may also contribute to hyperpigmentation [3, 38]. Whipple's disease could also rarely involve the oesophagus and large bowel [39]. Asymptomatic subjects with HIV infection might have unexpected colonisation of the lung by *Tropheryma whipplei*, which is reduced by effective antiretroviral therapy and merits further study for a potential pathogenic role in chronic pulmonary complications of HIV infection [40]. 

Moreover, besides classical Whipple's disease, there are newly recognised infections with *Tropheryma whipplei*, which do not fit in the concept of classical Whipple's disease, for example, acute self-limiting infection and isolated *Tropheryma whipplei* endocarditis [41]. 

### 5.2. Physical Findings

Kachexia (with oedema of the lower extremities) is a typical leading sign of advanced Whipple's disease. Physical findings include arthritis (90%), peripheral lymphadenopathy (up to 50%), low-grade fever (one third of cases), neurologic signs (up to 20%), polyserositis, uveitis, cheilosis, glossitis, purpura, and splenomegaly. Murmurs of aortic or mitral insufficiency may be found in about 25% of patients. In about 40% of patients, skin hyperpigmentation and hypotension can be also present. 

### 5.3. Laboratory Findings

Anaemia (usually microcytic or normocytic), relative lymphopenia, and increased erythrocyte sedimentation rate are consistent findings (75%). Laboratory markers of malnutrition are usually found (decreased total lymphocytes count, low serum albumin, prealbumin, and transferrin). Steatorrhoea is severe in advanced Whipple's disease (in over 90% of patients). D-xylose test is decreased (in 75% of patients). Secondary protein-losing enteropathy can be present. Because the disease primarily involves the proximal small intestine, B12 and bile acid malabsorption is uncommon. 

## 6. Diagnosis

**Figure 1:**
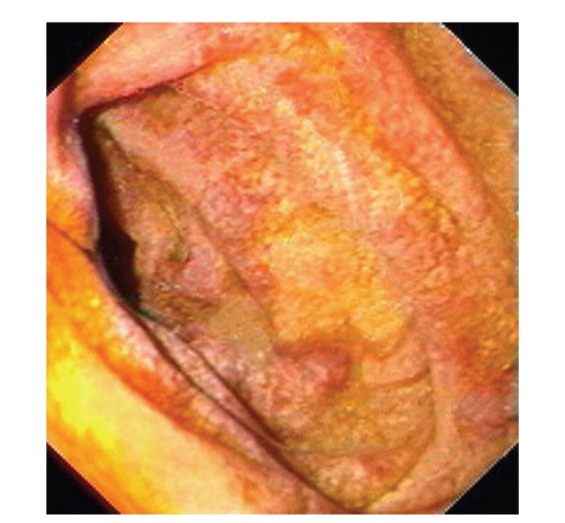
Whipple's disease: severe involvement of the distal duodenum (D4). Folds are low, and mucosa is swollen and grey-yellowish with multiple reddish spots (small mucosal haemorrhages).

**Figure 2:**
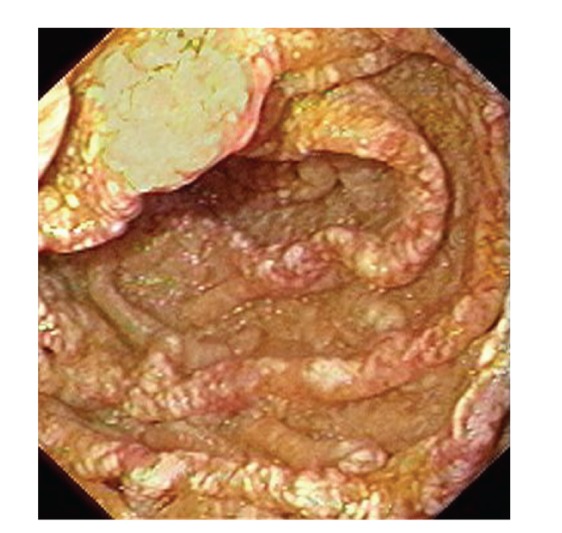
Whipple's disease: jejunal mucosa is swollen and grey-pink with small whitish areas and multiple tiny mucosal haemorrhages.

**Figure 3:**
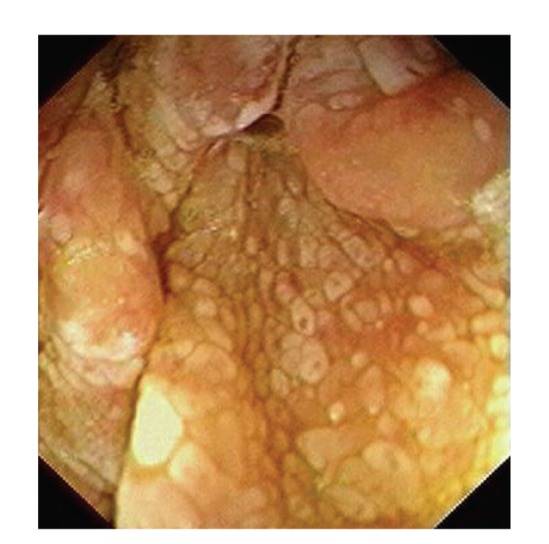
Whipple's disease: endoscopic view of the proximal jejunum. Characteristic whitish areas protrude a little the above surrounding relief.

**Figure 4:**
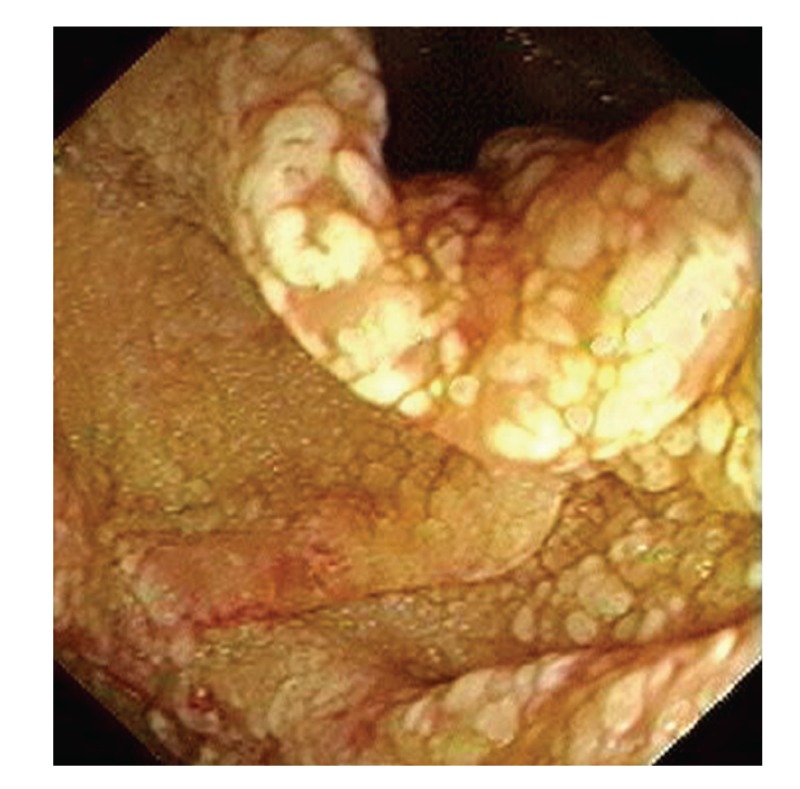
Whipple's disease: detailed view on whitish plaques on the top of fold.

**Figure 5:**
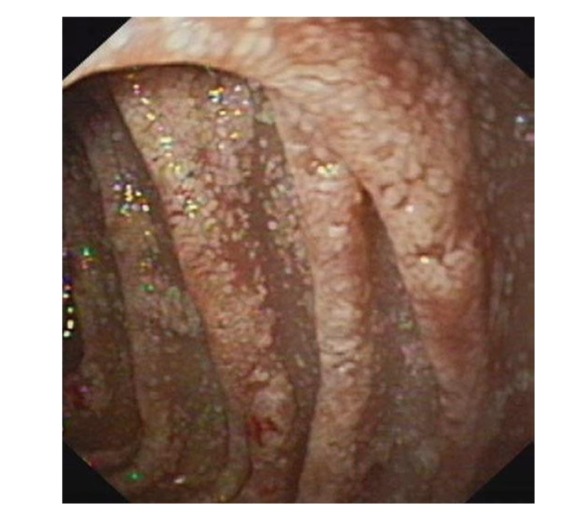
Whipple's disease: picture of the jejunum. Characteristic whitish areas protrude the above surrounding relief.

**Figure 6:**
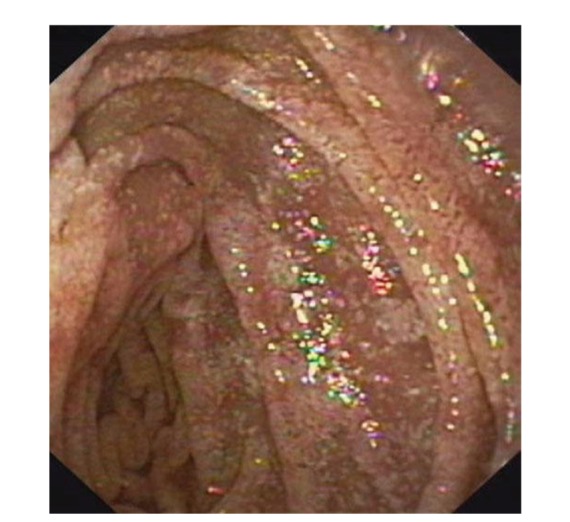
Whipple's disease: endoscopic view of the proximal jejunum. Transverse folds are low, reduced, and swollen.

**Figure 7:**
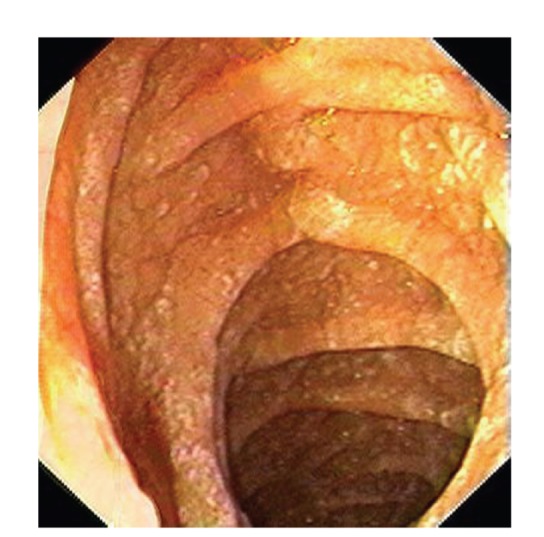
Whipple's disease: irregular rugged surface of the jejunal mucosa with an appearance like being dusted with flour.

**Figure 8:**
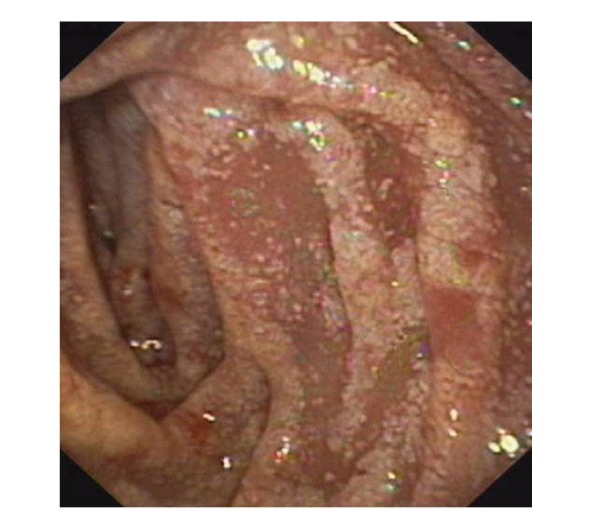
Whipple's disease: jejunal mucosa is grey-pink with small whitish areas.

**Figure 9:**
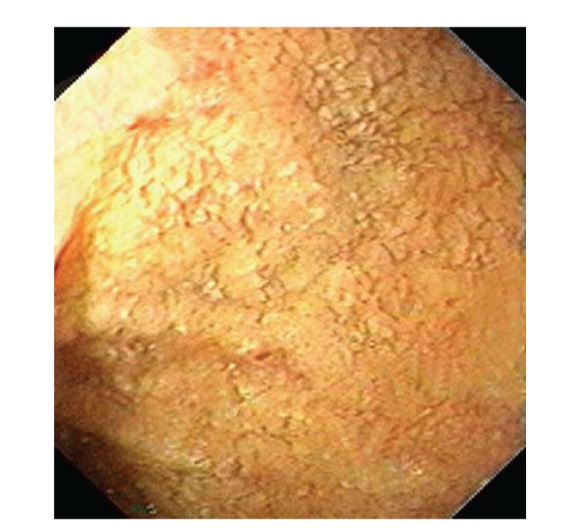
Whipple's disease: no folds are seen in the jejunum. Surface of mucosa is irregular; there are several tiny haemorrhages in mucosa with an appearance like being dusted with flour.

**Figure 10:**
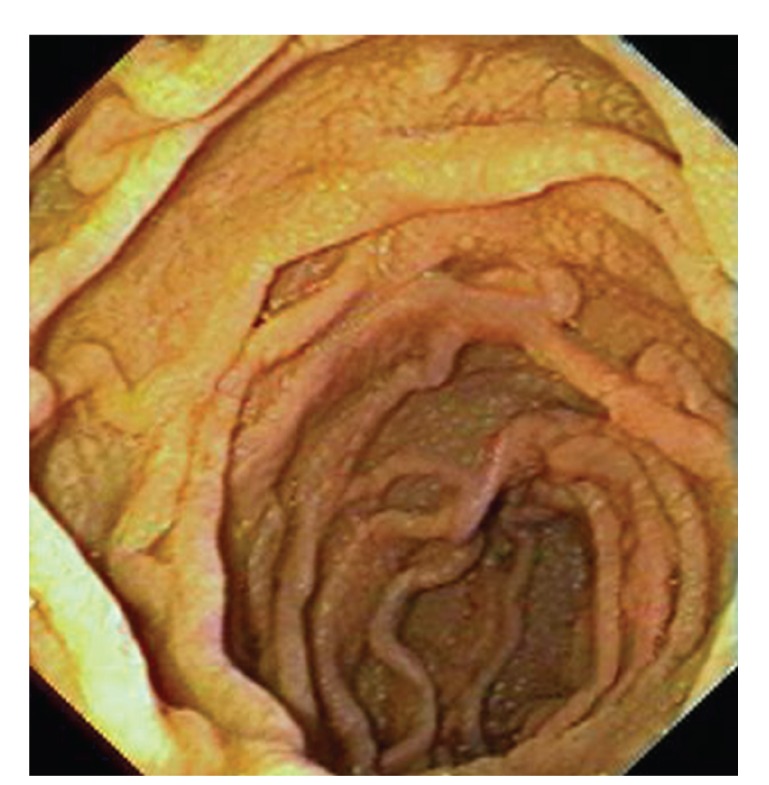
Whipple's disease: control enteroscopy after two-month treatment (with doxycycline and salazopyrine). Picture of the distal duodenum. Folds are still lower with chaotic disarrangement but macroscopically picture of mucosa has significantly improved.

**Figure 11:**
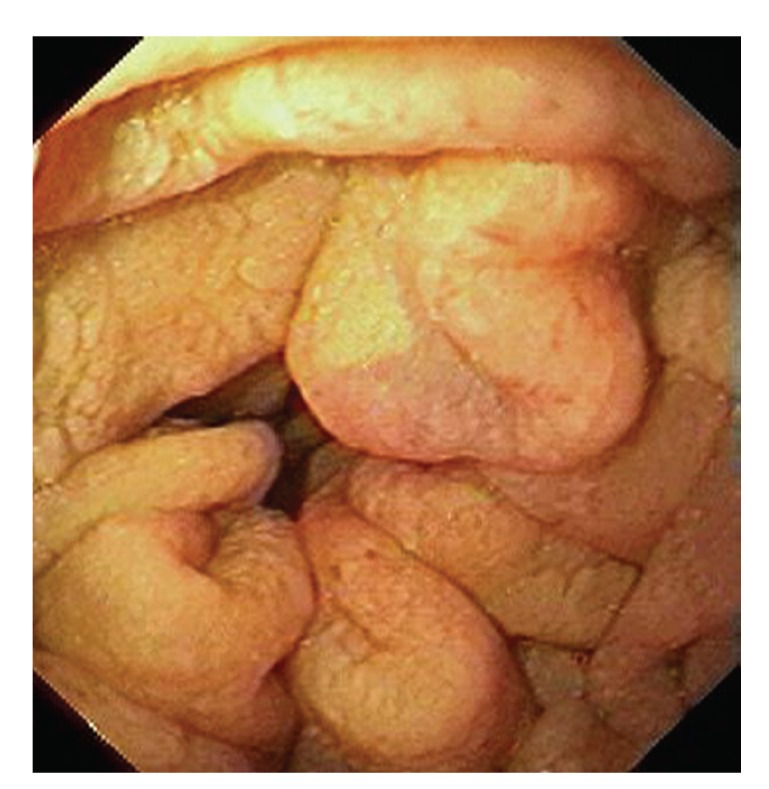
Whipple's disease: control enteroscopy after two-month treatment. Detail view of the jejunal mucosa. Mucosa is still swollen with tiny granular pattern.

**Figure 12:**
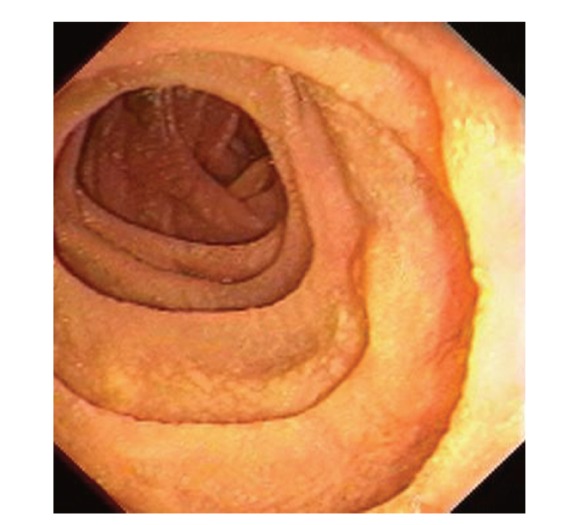
Whipple's disease: control enteroscopy after one-year treatment. Morphology of jejunal folds has normalised, surface is still rough, mucosa has fine granular pattern, and small areas of grey-yellowish colour still persist.

**Figure 13:**
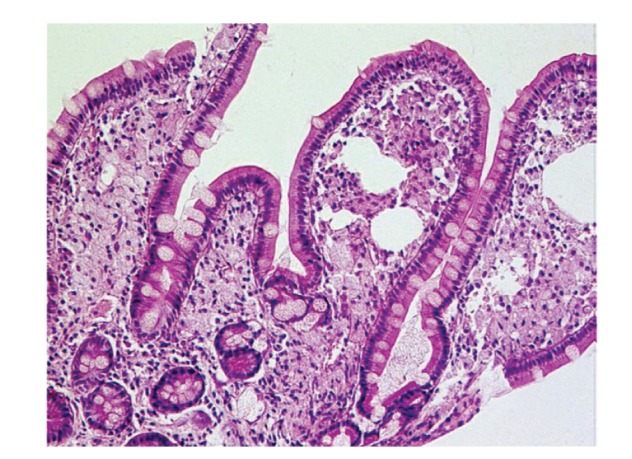
Whipple's disease: obvious enlargement of the villi filled by foamy macrophages. Hematoxylin-eosin staining. Courtesy of Jan Nožička, MD, PhD. Reproduced with permission from Bureš and Rejchrt [42].

**Figure 14:**
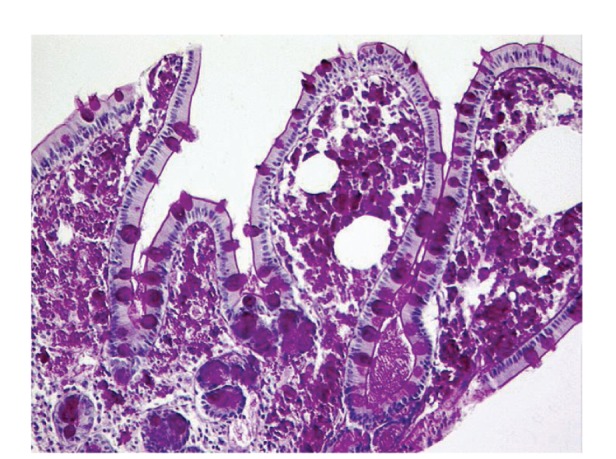
Whipple's disease: intensely PAS-positive macrophages occupying the lamina propria mucosae. Goblet cells are positively stained, too. Brush border of enterocytes is also marked (as a deep purple line). PAS staining. Courtesy of Jan Nožička, MD, PhD. Reproduced with permission from Bureš and Rejchrt [42].

**Figure 15:**
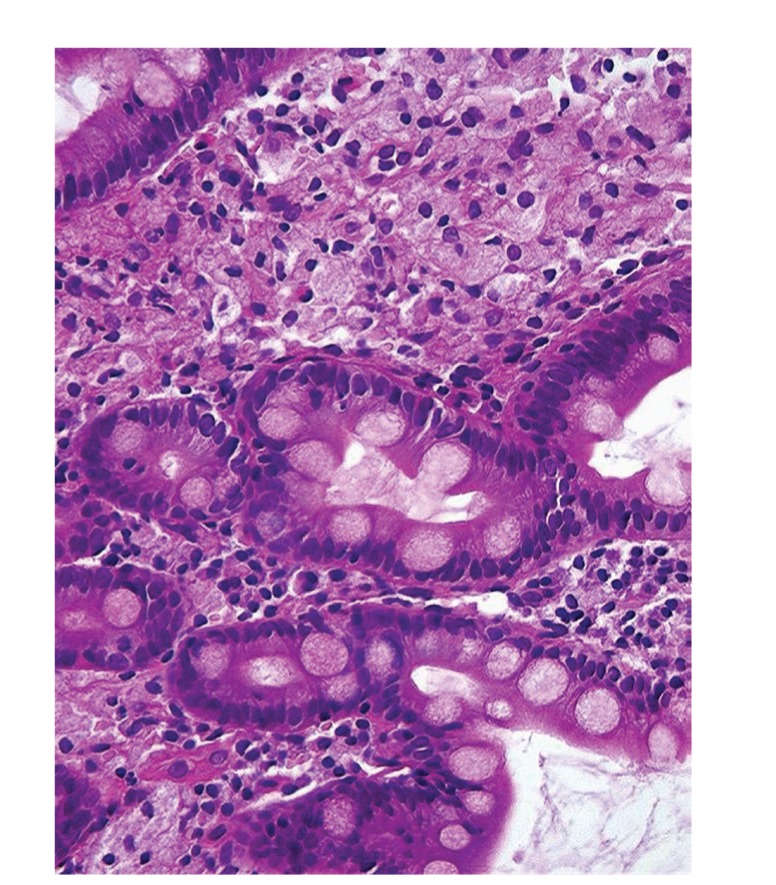
Whipple's disease: detailed view of macrophages in the lamina propria mucosae. Hematoxylin-eosin staining. Courtesy of Jan Nožička, MD, PhD. Reproduced with permission from Bureš and Rejchrt [42].

Upper gastrointestinal endoscopy is the diagnostic method of choice [42, 43]. Duodenoscopy (with biopsies) can already in its own right be diagnostic [42, 44–48]. Enteroscopy reveals a characteristic small intestinal pattern (see Figures 1, 2, 3, 4, 5, 6, 7, 8, 9, 10, 11, and 12 for details). Histology of biopsy specimens of the small bowel reveals macrophages containing periodic-acid-Schiff- (PAS-) positive material (large glycoprotein granules) and large lipid droplets in the lamina propria of small intestinal villi (see Figures 13, 14, and 15). Histology of the peripheral lymph node may also show foamy macrophages containing PAS-positive material. Similar PAS-positive macrophages may infiltrate many other organs, including the heart, brain, lung, spleen, liver, and pancreas. Electron microscopy studies can identify bacteria and characteristic lysosomes in intestinal histiocytes (see Figure 17). Wireless capsule endoscopy may help to clarify some obscure cases [49] with further recommendation for subsequent biopsy of small intestinal mucosa. Confocal laser endomicroscopy appearance of Whipple's disease was also described [50]. Fine-needle biopsy under endoscopic ultrasonic guidance may be successful in the diagnosis of Whipple's disease involving abdominal lymph nodes [51]. PCR techniques (of stools or saliva), bacteria culture, and tissue immunodetection of *Tropheryma whipplei* are not widely available yet. 

## 7. Differential Diagnosis

The differential diagnosis of Whipple's disease includes other malabsorptive diseases with diffuse small intestinal involvement (e.g., coeliac disease, tuberculosis, and *Mycobacterium avium* infection of the small bowel, histoplasmosis, non-steroidal anti-inflammatory drug-induced injury to the small bowel) and infiltrative diseases of the small intestine (e.g., non-Hodgkin lymphoma and amyloidosis).

Coexistence of diarrhoea, malabsorption, weight loss, seronegative arthritis, lymphadenopathy, and low-grade fever should alert the doctor to the possible diagnosis of Whipple's disease and lead to biopsy of the small intestinal mucosa. Other causes of malnutrition (mostly protein-energy malnutrition—kwashiorkor) must be excluded. 

In the case of pulmonary hypertension in Whipple's disease, primary pulmonary hypertension and other possible causes must be considered. Sometimes it is necessary to exclude connective tissue disease, inflammatory bowel disease with enteropathy-associated arthritis, hyperthyreosis, and AIDS. 

Addison's disease (primary adrenal cortical insufficiency) must be excluded in the case of orthostatic hypotension, skin hyperpigmentation, low serum natrium (hyponatremia), and high serum potassium (hyperkalemia). 

Fever of Whipple's disease can precede gastrointestinal symptoms by months; thus, the differential diagnosis of fever of unknown origin must also consider Whipple's disease. 

Timely diagnosis might be difficult in oligosymptomatic cases without gastrointestinal symptoms (15%). It is mandatory to properly evaluate possible psychiatric and neurologic signs (cognitive impairment, confusion, dementia, nystagmus, ophthalmoplegia, ataxia, muscle weakness, sensory loss, or posterior column signs) that can be uncommon signs of Whipple's disease. Central nervous system involvement is a particularly difficult problem because it may not respond to antibiotic therapy. 

In the histopathology of small intestinal biopsy specimens, other possible causes of PAS-positive staining of macrophages must be considered (e.g., *Mycobacterium avium* infection, systemic histoplasmosis, or macroglobulinemia) [52]. This might be difficult in some cases even in endoscopy as *Mycobacterium avium* infection might also mimic the enteroscopic pattern of Whipple's disease [53, 54]. 

## 8. Treatment

Whipple's disease was uniformly fatal prior to the use of antibiotics for its treatment. There is no general consensus concerning therapy for Whipple's disease. Several regimens were tried, including penicillin, amoxicillin-clavulanic acid, chloramphenicol, streptomycin and other aminoglycosides, macrolides, cephalosporins, tetracycline, doxycycline, trimethoprim-sulfamethoxazole, salazopyrine, and hydrochloroquine. *Tropheryma whipplei* is naturally resistant to fluoroquinolones [55]. 

Currently, most authors recommend starting with i.v. ceftriaxone (2 g once a day) or i.v. penicillin G (2–4 million units every four hours) for 2 to 4 weeks followed by peroral trimethoprim-sulfamethoxazole (960 mg twice daily) for one year. An alternative to trimethoprim-sulfamethoxazole is represented by doxycycline (100 mg twice daily p.o.) plus hydroxychloroquine (200 mg three times daily p.o.) for one year [3]. 

A large prospective trial (with 40 Central European patients from 2004 to 2008) compared the efficacy of ceftriaxone to meropenem for two weeks followed by oral trimethoprim-sulfamethoxazole for one year to prevent central nervous system manifestations. This study demonstrated very good response rates in both groups [56]. Recently, ceftriaxone followed by three months of trimethoprim-sulfamethoxazole was as efficacious as ceftriaxone followed by one-year trimethoprim-sulfamethoxazole in the treatment of Whipple's disease [57]. Human recombinant interferon gamma was successfully tried in a case of central nervous system involvement and refractory disease [58, 59]. 

## 9. Prognosis

When recognised properly and on time, Whipple's disease can usually be fully cured with long-term antibiotic therapy or long-lasting remission can be achieved. Extraintestinal symptoms often disappear within a few days, and gastrointestinal symptoms and malnutrition are resolved within two to three months. Neurologic manifestations of Whipple's disease have the most serious consequences with a decreased chance of full recovery. Patients with Whipple's disease do not appear to be prone to opportunistic infections or to malignancy. 

In general, prematurely ended treatment is associated with a 40% risk of relapse of the disease. Relapsing disease usually responds worse to the new series of therapy. Untreated Whipple's disease is ultimately fatal.

## 10. Our Own Experience 

**Figure 16:**
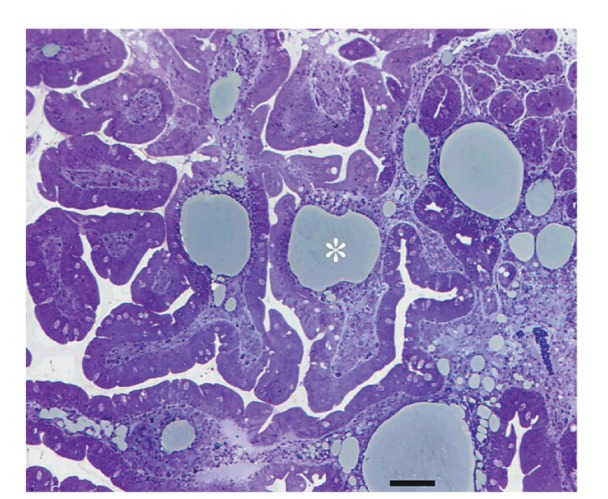
Accumulation of large lipid droplets (asterisk) in the lamina propria of intestinal villi. Semi-thin resin section, scale = 0.1 mm. Courtesy of Ladislav Kubeš, MD, PhD. Reproduced with permission from Bureš and Rejchrt [42].

**Figure 17:**
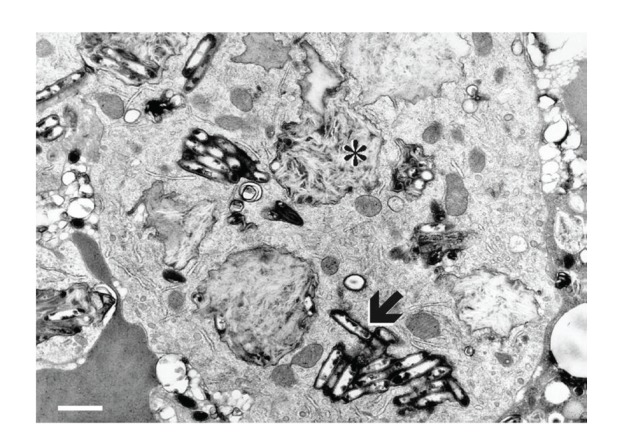
Groups of microbes (arrow) and characteristic lysosomes (asterisk) present in an intestinal histiocyte in Whipple's disease. Electron micrograph, scale = 2 *μ*m. Courtesy of Professor Josef Špaček, MD, DSc. Reproduced with permission from Bureš and Rejchrt [42].

**Figure 18:**
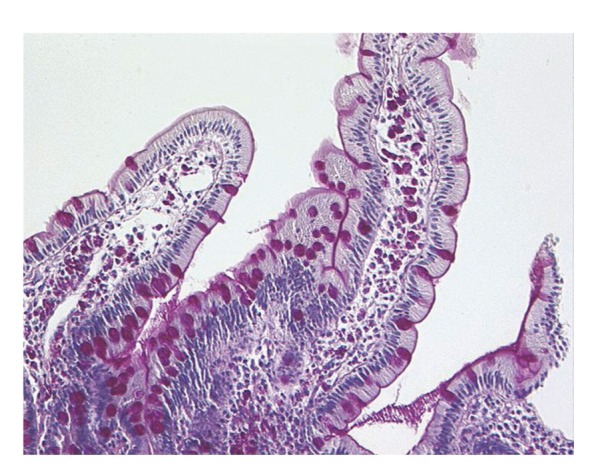
Whipple's disease: partial disappearance of PAS positive macrophages from the lamina propria mucosae following the therapy. PAS staining. Courtesy of Jan Nožička, MD, PhD. Reproduced with permission from Bureš and Rejchrt [42].

We recorded five cases of Whipple's disease in the period 1994–2013 at our Department, a single tertiary centre, where 7-8 thousand of GI endoscopies are performed per year. All patients were Central European Caucasians. Four persons were newly diagnosed, and one subject (patient number 5) was referred to our Department because of the second relapse of disease (35 years after the first onset); see Table 1 for details. Nobody was presented with neurological symptoms. All patients had a characteristic endoscopic pattern of the small intestinal mucosa and typical histopathology findings (see Figures 1, 2, 3, 4, 5, 6, 7, 8, 9, 10, 11, 12, 13, 14, 15, 16, 17, and 18). The outcome was excellent. All patients achieved full clinical, laboratory, and endoscopic remission after one year of treatment with doxycycline and salazopyrine. Remarkable increase of body weight was achieved in all our five patients (a gain from 10 kg up to 53 kg within one year). One patient improved his metabolic bone disease substantially within a one-year period (a bone mass gain by 19%). Two subjects were subsequently lost from our follow-up.

## 11. Discussion

Whipple's disease has been recognised as a bacterial disease. The first noncontroversial identification of the organism that causes Whipple's disease was provided by Wilson et al. in 1991 [60] and was based on partial 16S rRNA gene amplification and sequencing from a single patient. The causative agent was established successfully in vitro in 1999 [6]. 

Whipple's disease is a rare disease. We have recorded only five cases at our tertiary gastroenterology centre within a 20-year period (no new case during the last 8 years). Patients were presented mostly with weight loss, diarrhoea, microcytic anaemia, and arthralgias. We decided on i.v. penicillin G or amoxicillin-clavulanic acid + i.v. gentamicin for two weeks, followed by p.o. doxycycline (100 mg per day) plus p.o. salazopyrine (3 g per day) for 1 year. Full remission was achieved in all our patients. We do not recommend streptomycin because of the significant risk of serious side effects of this treatment. We consider salazopyrine a suitable alternative to trimethoprim-sulfamethoxazole. The susceptibility of bacteria to trimethoprim-sulfamethoxazole is due to sulfamethoxazole alone [61]. None of our patients were presented with neurologic symptoms. 

## 12. Conclusions

Whipple's disease is chronic infectious systemic disease caused by the bacterium *Tropheryma whipplei*. Nondeforming arthritis is frequently an initial complaint. Gastrointestinal and general symptoms include marked diarrhoea (with serious malabsorption), abdominal pain, prominent weight loss, and low-grade fever. Possible neurologic symptoms (up to 20%) might be associated with worse prognosis. Diagnosis is based on the clinical picture and small intestinal histology revealing foamy macrophages containing periodic-acid-Schiff- (PAS-) positive material. Long-term (up to one year) antibiotic therapy provides a favourable outcome in the vast majority of cases.

## Figures and Tables

**Table 1:** Characteristics of five patients with Whipple's disease diagnosed in the period 1994–2013.

Patient	Gender	Age at the diagnosis (time to diagnosis)	Clinical symptoms	Treatment	Outcome*
# 1	Male	49 years (3 years)	Weight loss (20 kg); microcytic anaemia; metabolic bone disease	i.v. penicillin G + i.v. gentamicin for two weeks; p.o. doxycycline (100 mg per day) + p.o. salazopyrine (3 g per day) for 1 year	Full remission

# 2	Male	53 years (12 months)	Weight loss (11 kg); diarrhoea; microcytic anaemia; headache; dyspnoea; orthostatic hypotension; skin rash	i.v. amoxicillin-clavulanic acid + i.v. gentamicin for two weeks; p.o. doxycycline (100 mg per day) + p.o. salazopyrine (3 g per day) for 1 year	Full remission

# 3	Male	44 years (6 months)	Weight loss (18 kg); diarrhoea; severe malabsorption; severe kwashiorkor-type malnutrition; metabolic bone disease	i.v. amoxicillin-clavulanic acid + i.v. gentamicin for two weeks; p.o. doxycycline (100 mg per day) + p.o. salazopyrine (3 g per day) for 1 year	Full remission

# 4	Female	58 years (6 months)	Arthralgias; weight loss (10.5 kg); diarrhoea; malnutrition; microcytic anaemia	i.v. amoxicillin-clavulanic acid + i.v. gentamicin for two weeks; p.o. doxycycline (100 mg per day) + p.o. salazopyrine (3 g per day) for 1 year	Full remission

# 5	Female	24 years** (12 months)	Arthralgias; peripheral and abdominal lymph nodes; muscle weakness; fatigue	i.v. penicillin G + i.v. gentamicin for two weeks; p.o. doxycycline (100 mg per day) + p.o. salazopyrine (3 g per day) for 1 year	Full remission

Notes. *Clinical, endoscopic, and laboratory outcome after one year of treatment.

**Patient no. 5: a 59-year-old female was referred to our University Department because of the second relapse of Whipple's disease after 18 years (the first onset at 24 and the first relapse at 41 years).
